# The Impact of Transformational Leadership on Service Employees in the Hotel Industry

**DOI:** 10.3390/bs13090731

**Published:** 2023-08-31

**Authors:** Jin-Kyu Kim, Jae-Jang Yang, Yong-Ki Lee

**Affiliations:** 1 Graduate School of Business, Sejong University, Seoul 05006, Republic of Korea; leadership81@naver.com; 2 School of Business and Graduate School of eMA, Sejong University, Seoul 05006, Republic of Korea; doublej@sejong.ac.kr; 3 School of Business and Sejong Carbon Neutral ESG Institute, Sejong University, Seoul 05006, Republic of Korea

**Keywords:** transformation leadership, organizational identification, creativity, task performance, Korean hotel industry

## Abstract

Transformational leadership is important to the hotel industry where employees play a critical role in delivering the service. Transformational leadership is a leadership style that causes changes in employees by sharing the organization’s vision and goals, providing necessary resources, promoting intellectual stimulation, and expecting high performance. This study uses four dimensions of transformational leadership to investigate how the dimensions influence employees’ organizational identification, creativity, and task performance. In order to test the proposed model, data were gathered from employees of six hotels in South Korea. The data were analyzed with SmartPLS 4.0 program. The finding shows that four dimensions of transformational leadership have different effects on organizational identification and creativity. While core transformational leadership and supportive leader behavior enhance employees’ organizational identification, which affects creativity and task performance, intellectual stimulation has an impact on creativity, which influences task performance. The post hoc analysis shows that organizational identification fully mediates the relationship between two dimensions of transformational leadership and creativity. This study contributes to the existing literatures of leadership theory and social identification theory by expanding the knowledge on the role of transformational leadership on employees. Some theoretical and practical implications are offered.

## 1. Introduction

Transformational leadership is a style of leadership that inspires followers to change their expectations, perceptions, and behaviors to work toward a common goal [1]. Simply put, it focuses on transforming followers for the betterment of organization. Because employees play a critical role in determining customers’ perception of the service, having high-performing employees is important for achieving the organization’s goals [2]. Transformational leadership can influence employees in terms of how they feel about the organization, perceive the job, and perform [3]. Faced with multitudes of challenges post- COVID-19 pandemic, the hotel industry needs to find ways to engage employees in the job and deliver high-quality services [4]. Transformational leadership may offer a solution to the problems by influencing the employees [5,6,7]. Prior research suggests that transformational leaders possess four characteristics of leadership behaviors: (a) core transformational behavior, (b) high-performance expectation, (c) supportive leader behavior, and (d) intellectual stimulation. This study uses four dimensions to examine how they affect employees in terms of their sense of belongingness (organizational identification), creativity, and task performance [8,9,10].

There are some studies that examined transformational leadership in the hotel context. However, none of them considered the effect of transformational leadership on employees’ organizational identification, creativity, and job performance simultaneously. Given that hotel employees play a critical role in in determining the quality of service [11], it is important to understand how different dimensions of leadership influence the employees. Recent studies suggest that employees’ performance is directly related to the organization’s success [12], and employees’ sense of belongingness to the organization (organizational identification) [13] and creativity are important for enhancing the organization’s performance [14]. Therefore, this study examines the role of organizational identification and creativity in affecting employees’ task performance [15,16].

One of the unique contributions of this study is that it uses four dimensions of transformational leadership to understand their different effects on the employees. Most previous studies in the hotel industry treated transformational leadership as a single dimension [17,18]. Because transformational leadership is a complex concept that includes a leader’s personal characteristics [19] and management style, there are limitations to studying it as a single dimension. Therefore, this study uses four dimensions based on prior research. Another unique aspect of this study is that it examines leadership of executive leaders (general managers). Prior research focused on dyadic relationships between direct supervisors (e.g., [20,21]) and employees. This study is an attempt to address the gap in the literature by examining employees’ perception of the executive leader and its effect on the employees. The findings will add evidence to the leadership literature. Lastly, this study, by examining the intricate relationship among leadership, employees’ organizational identification, creativity, and job performance, presents transformational leadership style as an important consideration for making changes for employees and the organization. The study’s findings will serve as a valuable guide for managers who constantly invest their managerial and human resources in stimulating creativity and enhancing employee job performance.

This study will specifically address the following research questions: (1) How does transformational leadership style influence employees’ organizational identification and creativity? (2) Which leadership dimensions are more effective at bringing out different employee-related outcomes? (3) Does organizational identification have an impact on creativity? and (4) Are organizational identification and creativity helpful for enhancing employees’ job performance? Gaining an understanding of the influence of transformational leadership style on employees will be strategically important. This understanding will help leaders focus on certain aspects of the leadership behaviors to achieve the desirable outcomes. In addition, the study will unravel the complex relation among organizational identification, creativity, and task performance, and offer implications on how to help employees succeed in their job.

The structure of this paper is as follows. First, we discuss the literature and formulate hypotheses that address the study’s objectives. Subsequently, we outline the methodology, including the sampling process, data collection, and measures used. Finally, we report the data analyses, offer discussions of the results, recognize the study’s limitations, and make suggestions for future studies.

## 2. Literature Review

### 2.1. Transformational Leadership

The concept of transformational leadership was first proposed by Downton in 1973 [22]. Burns later solidified the concept by comparing transformational leadership with transactional leadership [23]. Because employees’ work-related behavior is related to the organization’s financial performance, organizations must pay attention to the behaviors of employees [24]. Pradhan and Jena [25] revealed that transformational leadership allows employees to continue to participate in their work to achieve goals and promotes innovative behavior. Since leadership is essential for improving the organization’s performance (e.g., [26,27]), many researchers have investigated how transformational leadership would affect employees’ attitude and behavior. For example, Eliyana and Ma’arif [28] show that transformational leadership helps increase employee productivity. Similarly, Alrowwad et al. [29] find that transformational leadership increases employee innovation, which in turn improves organizational performance. Also, Ng [30] reports that transformational leadership affects employee performance.

While some studies treated transformational leadership as a single dimension (e.g., [29]), some researchers suggest that it is comprised of four dimensions: core transformational leader behavior, high-performance expectations, supportive leader behavior, and intellectual stimulation (e.g., [6,7]).

#### 2.1.1. Core Transformational Leader Behavior

Core transformational leader behavior refers to behaviors in which leaders clarify the vision to employees, provide appropriate success models, and support the employees to achieve the organizational goals [31,32]. Transformational leaders articulate a vision for the organization [33], sets organizational goals, and make effective communication to the employees. Transformational leaders will motivate the employees to work hard and become committed to the organization. Ultimately, successful transformational leaders will make the employees do more beyond their job demand [34]. Core transformational leaders act to be themselves and become role models for their employees [35]. Through core transformational behaviors, leaders will gain trust and respect from the employees, and employees will follow and emulate the leader [36].

#### 2.1.2. High-Performance Expectations

Another element of transformational leadership is high-performance expectation. Transformational leaders set high expectations on job performance and provide tools and resources necessary for the employees to utilize [7,37]. When leaders expect high job performance, employees tend to hold high expectations of themselves (i.e., belief in their ability to effectively complete a given task) [38].

Based on the LMX theory (Leader–Member Exchange Theory), the relationship between leaders and members within an organization affects performance of the individuals and the organization [39]. According to the theory, when a leader expresses his/her expectation that employees would or should deliver high job performance, the employees respect and trust the leader and commit their own resources to performing the job [40]. This high-performance expectation that leaders have of employees is expected to be directly related to job performance [41].

#### 2.1.3. Supportive Leader Behavior

Supportive leader behavior refers to a leader’s behavior that is helpful for employees to complete a given task (e.g., encouraging employees to make improvements through developmental programs) [39]. It also refers to the extent to which leaders are actively involved in resolving difficult situations for the employees [42]. According to the Path–Goal Theory, leaders who are supportive and helpful can make subordinates improve their job performance and be satisfied with the organization [43]. Leaders need to engage in behaviors that address the needs of the employees. This will create a work environment where employees are motivated to achieve a high level of job performance and overcome obstacles [44,45].

#### 2.1.4. Intellectual Stimulation

Intellectual stimulation refers to a leader’s behavior that encourages employees to think creatively to solve problems on their own and promotes learning [46]. Leaders practicing intellectual stimulation allow employees to break away from stereotypes and come up with new ideas to solve a problem [47]. Intellectual stimulation will play an important role in encouraging employees to engage in organizational learning [48]. Leaders may stimulate intellectual thinking by constantly questioning or challenging employees’ thought processes, ultimately leading to employees’ involvement in idea generation and implementation of the ideas [49]. Intellectually stimulating leaders can inspire and motivate employees because they encourage employees to develop themselves, find solutions to problems in new ways, and become better at the job [50,51].

### 2.2. Organizational Identification

Organizational identification is an employee’s sense of identity in relation to the organization to which he or she belongs [52,53]. Based on social identity theory, organizational identification is a sense of belongingness and emotional attachment to the organization [54]. Social identity theory posits that an individual’s self-perception is built based on his/her identification with the group he/she belongs to [13,46,55]. Organizational identification, which is a psychological connection between an organization and its employees, is likely to affect the employees’ behavior. When employees identify themselves in association with the organization, they are likely to be genuinely interested in the success of the organization, help coworkers, go extra miles for the customers, and follow the organization’s policies and rules. The importance of organizational identification is more pronounced in the hospitality industry because employees play a critical role in delivering services and determining customers’ experience with the organization [56,57].

### 2.3. Creativity

Creativity is defined as the ability to create something new with elements that already exist (i.e., ability to create meaningful new combinations) or the ability to generate new ideas for problem-solving [58,59]. Creativity is a unique ability that people have, and it entails a creative thinking process [60]. Afsar et al. [61] suggested that the best way to stimulate innovation in an organization is to promote creativity of its members. When employees share useful new ideas, it can bring about changes to products and services [47]. Therefore, leaders should value creative work and support the culture where innovation is appreciated [35]. Transformational leaders who value creativity will need to stimulate employees to come up with fresh ideas.

### 2.4. Task Performance

Employee task performance is directly related to the organization’s performance and gives the organization a competitive advantage [62]. Task performance refers to the quality and quantity of work based on the job description [57]. Task performance is an essential precedent for achieving goals for the employees and the organization [63,64]. Employee task performance can be enhanced when employees perform the given task effectively and efficiently (speed and accuracy), which will have a direct impact on the organization’s overall performance [12]. Thus, a high level of employee engagement is necessary to promote task-related performance, which ultimately leads to the organization’s competitive advantage [65]. Leadership style has a significant impact on employees’ task performance when the leader encourages employees to continuously develop their job skills and gain knowledge [66]. Employee task performance is found to increase when employees are encouraged to exercise their creative and innovative abilities [67,68].

## 3. Hypotheses

### 3.1. The Impact of Transformational Leadership on Organizational Identification

Based on previous studies, this study examines four dimensions of transformational leadership and their effect on employees. Transformational leaders share a vision and set goals for the organization. When the leaders provide appropriate support and resources needed, the employees will feel supported, develop a bond with the organization, and identify themselves with the organization [69,70,71].

Leaders who hold high-performance expectations will make employees conscious of their performance goals and put extra efforts to achieve the goals. Based on previous research [72,73,74], leaders’ high-performance expectations increase employees’ job engagement and likelihood to achieve their goals. When employees feel that the leaders expect them to perform at a high level, they are likely to make conscious efforts. When they make efforts and achieve the goals, they will be satisfied with their job and the organization, which will increase their likelihood to identify themselves with the organization.

Leaders who are supportive will recognize employees’ individual needs, offer support and resources, and build a strong interpersonal relationship with the employees. Because employees will feel that they are supported by the leaders, they will feel empowered and develop a bond with the organization [75]. Prior research suggests that employees form an attachment and emotional connection to the organization when a leader displays empathy and offers support to the employees, especially when they are in a problematic situation [76].

Leaders who intellectually stimulate employees are likely to have a positive impact on the employees. When leaders create and nurture a culture that allows employees to try an experimental approach, the employees are likely to come up with new ideas [77]. When employees are allowed to share their perspectives and ideas and contribute to improving the organization’s performance, they will feel they are part of the organization and develop a bond with the organization [70,78,79]. In summary, this study proposes that the four dimensions of transformational leadership will have a positive impact on the employees (i.e., organizational identification). The following hypotheses are presented.

**H1.**  *Transformational leadership has a positive effect on the employees’ organizational identification*.

**H1-1.**  *Core transformational leader behavior has a positive effect on the employees’ organizational identification*.

**H1-2.**  *High-performance expectations have a positive effect on the employees’ organizational identification*.

**H1-3.**  *Supportive leader behavior has a positive effect on the employees’ organizational identification*.

**H1-4.**  *Intellectual stimulation has a positive effect on the employees’ organizational identification*.

### 3.2. The Impact of Transformational Leadership on Creativity

Transformational leadership is closely related to employees’ creativity at the individual level [47,80,81]. Transformational leaders encourage employees to achieve their goals by sharing the vision and using motivation tactics [82]. Therefore, employees tend to freely express and implement their novel ideas [83]. Leaders’ high-performance expectations energize employees to achieve work goals [84]. This makes employees feel challenged and energized to approach new ways in their work, influencing creativity [35].

Transformational leaders show empathy toward employees and provide support so that the employees can perform tasks and achieve their goals [39]. Leaders who intellectually stimulate employees by challenging old ways will create a work environment where employees feel comfortable with thinking new innovative ways [47,77,80]. Prior research suggests that transformational leaders stimulate employees to be creative, and this helps the organization become innovative and sustainable [85]. This study hypothesizes that the four dimensions of transformational leadership will have a positive effect on employees’ creativity.

**H2.**  *Transformational leadership has a positive effect on the employees’ creativity*.

**H2-1.**  *Core transformational leader behavior has a positive effect on the employees’ creativity*.

**H2-2.**  *High-performance expectations have a positive effect on the employees’ creativity*.

**H2-3.**  *Supportive leader behavior has a positive effect on the employees’ creativity*.

**H2-4.**  *Intellectual stimulation has a positive effect on the employees’ creativity*.

### 3.3. The Impact of Organizational Identification on Creativity

Organizational identification is anticipated to positively impact employees’ creativity through their sense of belonging to the organization and perception of their role [86]. Organizational identification is an important factor for maintaining a long-term relationship between employees and the organization [87]. It is found to be an important variable in explaining and predicting employees’ attitudes and behaviors in the organization [88]. Employees who identify themselves with the organization are likely to link their careers with the organization’s future and view the organization’s interests as their own [89]. These employees are more likely to put extra efforts into job performance to improve organizational effectiveness [87]. Because they are emotionally engaged in the organization, they are likely to share their creative ideas and solutions to the problems [90]. Liu et al. [91] discover that employees who possess a strong sense of organizational identification tend to synchronize their personal objectives with those of the organization, resulting in an increased display of creativity. Moreover, Kesen [92] shows a positive correlation between employees’ identification with the organization and their level of creativity, demonstrating that a higher level of identification leads to greater manifestation of creative behaviors, which include the generation of innovative ideas. Based on these preceding studies, the following hypothesis is presented.

**H3.**  *Employees’ organizational identification has a positive effect on creativity*.

### 3.4. The Impact of Organizational Identification on Task Performance

Organizational identification is expected to positively affect employees’ task performance [93]. This is because when employees perceive they are part of the organization, they are likely to work hard to achieve the organization’s goals [93,94]. In addition, the employees who work hard are more likely to receive support and resources from the organization, which positively impacts their task performance [95]. According to social identity theory [96], employees with a high degree of organizational identification are likely to be more active in activities that benefit the organization and put more efforts into improving the task performance [97]. Liu et al. [97] suggest that employees who identify themselves with the organization show a higher level of job performance than those who do not because they accept the organization’s goals as their own goals [98]. Thus, task performance of the employees is expected to be influenced by their organizational identification. The following hypothesis is presented.

**H4.**  *Employees’ organizational identification has a positive effect on task performance*.

### 3.5. The Impact of Creativity on Task Performance

Many studies [99,100,101] on innovation emphasize the importance of creativity. Creative solutions and ideas help organizations solve problems in an efficient way and move forward with new strategies. When employees are allowed to be creative, they are invested in the process of finding solutions and making changes for the organization. In addition, they can do the job more effectively because they have the creative approaches and solutions. For example, Ismail et al. [94] found that when an organization allows employees to engage in creative behavior, they are engaged in the organization and their creativity increases, which can improve job performance. Luthans et al. [100] and Semedo et al. [101] also found that creative people produce better outcomes than other peers by pursuing challenges and meeting goal settings. Therefore, this study hypothesizes that creativity has a positive impact on task performance.

**H5.**  *Creativity has a positive effect on task performance*.

**Figure 1:**
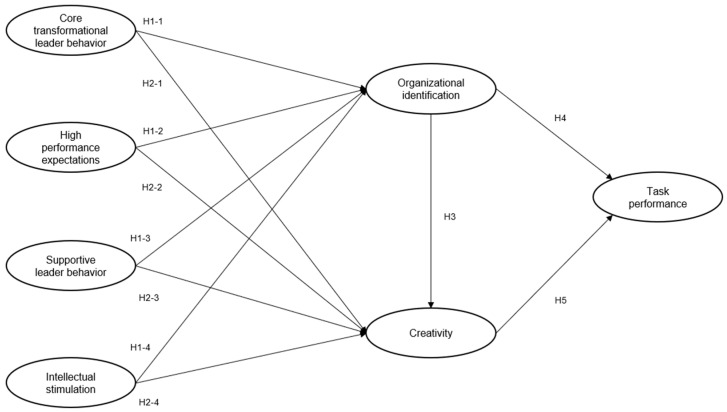
Proposed model.

Based on the hypotheses, the proposed model is shown in Figure 1.

## 4. Methodology

### 4.1. Measures

All items were measured with multiple items adopted from previous studies. The measures were anchored by 1 (strongly disagree) and 7 (strongly agree). Transformational leadership consisted of four dimensions including core transformational leader behavior (three items), high-performance expectations (two items), supportive leader behavior (five items), and intellectual stimulation (four items). These measures were adopted from the Lee et al.’s [7] study. Organizational identification was measured with four items adopted from the Amundsen’s [102] study. The study used four items to measure creativity and they were adopted from the Tsai et al. [103]’s study. Finally, task performance was measured with five items adopted from the Yang et al. [104]’s study.

### 4.2. Data Collection and Sampling

Data were gathered from employees of six luxury hotels in South Korea. All hotels had indigenous general managers. These hotels were chosen because they were known to use advanced management techniques and knowledge management systems [105].

Questionnaires accompanied by a cover letter were distributed to the hotels upon their agreement to participate in the study. The general managers encouraged their non-supervisory staff to complete the survey. In order to maintain privacy and minimize biases, we followed the survey administration procedure outlined in the study of Lee et al. [105]. Employees were asked to complete the survey and place it in a sealed envelope, which was then collected and returned to us. Out of the 600 questionnaires distributed to the six hotels managed by local GMs, we received 490 responses, resulting in a response rate of 81.7%. From these, we discarded 36 incomplete responses. After eliminating 36 incomplete responses, we coded and analyzed 454 questionnaires with an effective response rate of 75.7% for further analysis. A pre-test was carried out on 20 employees of two hotels to identify any potential biases or ambiguities. The survey questionnaire was modified based on the feedback received. To accurately translate the questionnaire from English to Korean, a back-translation process was employed. Three bilingual professors from Korea and one from the U.S. conducted three rounds of translation between Korean and English.

## 5. Results

### 5.1. Profile of the Respondents

The respondents’ profile is presented in Table 1. Gender composition is as follows: 53.3% for males and 46.7% for females. As for marital status, the number of unmarried (51.5%) was slightly higher than that of married (46.5%). The most common age group was 30 s (48.9%), followed by 20 s (32.2%) and 40 s (16.3%). In terms of education, respondents with a four-year college degree or above accounted for the largest proportion (71.8%) of the sample. Regarding job position, staff (52.9%) accounted for more than half, followed by managers (31.5%). More than half of the respondents (57.0%) worked five years or more and 43% of them worked four years or less.

### 5.2. Measurement Model

The measurement model and structural model were assessed with SmartPLS 4, which is a suitable tool for capturing the variation in exogenous variables [106,107,108]. PLS (Partial Least Squares) is an analytical method that is well-suited for assessing the explanatory power and predictive fit of models, with the objective of maximizing variance and minimizing structural errors [108,109].

Table 2 illustrates that the values of Cronbach’s alpha and composite reliabilities (CR) were found to exceed the threshold of 0.7, indicating a high level of internal consistency reliabilities. Convergent validity was established as the average variance extracted (AVE) values exceeded the acceptable threshold of 0.5.

Discriminant validity was well established, because the square root of AVE in each latent variable was larger than other correlation values among the latent constructs (see Table 3).

In addition, the Heterotrait–Monotrait (HTMT) ratio of correlations [110] values were under 0.085 (see Table 4).

### 5.3. Common Method Bias Assessment

In accordance with the procedures outlined in the study of Kang et al. [111], this study employed a combination of procedural and full collinearity test (simultaneous assessment of both vertical and lateral collinearity) to address common method biases. The procedural method encompassed the following steps. First, certain words and sentences that respondents found challenging to comprehend were identified and modified based on the results of the pre-test. Second, participants were informed about the research objectives and provided with instructions on how to complete the survey. Third, the questionnaire was designed in such a way that independent and dependent variables were not presented consecutively, preventing respondents from making inferences about the relationships between variables. Additionally, this study employed a statistical approach by examining the variance inflation factor (VIF) values, as established in previous research [112]. The analysis of VIF values indicated that common method bias was not a concern, as the values were below 3.3 (ranging from 1.646 to 2.961).

### 5.4. Structural Model Assessment

To ensure that multicollinearity was not a concern, we assessed the variance inflation factor (VIF) values, which were found to be below 3.3, indicating the absence of multicollinearity. The predictive power of the model was evaluated using the R^2^ values, which exceeded 29% [113]. According to Chin’s guideline [108], an R^2^ value of 0.67 indicates a strong explanatory power, 0.33 represents a medium level, and 0.19 indicates a weak level. Our study’s models demonstrated values higher than 0.29, indicating that there is a no problem in the evaluation of the structural model. The predictive relevance of the endogenous constructs was established by examining the Stone–Gesser (Q^2^) values, which represent the cross-validated redundancy. These values were greater than zero [113]. Lastly, the values of the standardized root mean square residual (SRMR) were examined. As these values were below 0.050 [114], the model fit was deemed acceptable.

### 5.5. Hypotheses Testing

**Figure 2:**
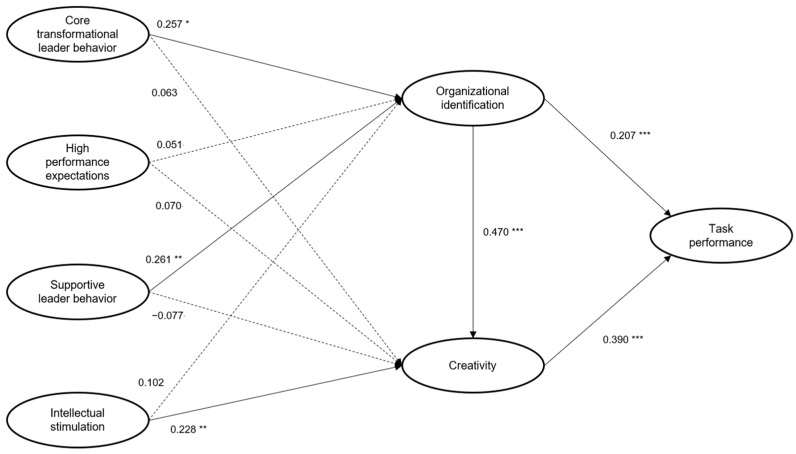
Estimates of the model (PLS). Note: * *p* < 0.10, ** *p* < 0.05, *** *p* < 0.01. Solid line = significant paths, Dotted line = non-significant paths.

H1 addressed the impact of four dimensions of transformational leadership on employees’ organizational identification. As shown in Figure 2 and Table 5, the finding shows that core transformational leadership behavior and supportive leader behavior significantly influence organizational identification. Therefore, H1-1 and H1-3 are supported. However, high-performance expectations and intellectual stimulation are not found to have a significant effect on organizational identification. Hence, H1-2 and H1-4 are not supported.

H2 was concerned with the impact of four dimensions of transformational leadership on creativity. The finding shows that intellectual stimulation significantly influences creativity. However, core transformational leadership behavior, high-performance expectations, and supportive leader behavior are not found to have a significant effect on creativity. Therefore, H1-4 is supported, while H1-1, H1-2, and H1-3 are not supported.

H3 and H4 predicted the impact of organizational identification on creativity and task performance. The finding shows that organizational identification significantly influences both creativity and task performance, supporting H3 and H4. Lastly, H5 addressed the impact of creativity on task performance. The finding shows that creativity significantly influences task performance. Therefore, H5 is supported.

### 5.6. Mediating Test

Additional analyses related to indirect effects were conducted to find out (1) whether organizational identification and creativity play a mediating role in the relationship between four dimensions of transformational leadership and task performance, and (2) whether creativity plays a mediating role in the relationship between organizational identification and task performance (see Table 6). We used the Sobel test for this purpose [115,116]. As shown in Table 6, only two dimensions of transformational leadership are mediated by organizational identification in their effects on creativity: core transformation leadership (γ_CTL→OI→CRE_; 0.121, *p* < 0.001; Z = 3.572, *p* < 0.001) and supportive leader behavior (γ_SLB→OI→CRE_; 0.123, *p* < 0.01; Z = 2.994, *p* < 0.01). In other words, organizational identification plays a full mediating role in the relationship between (a) core transformation leadership and creativity, and between (b) supportive leader behavior and creativity. In addition, Table 6 shows that creativity plays a partial mediating role in the relationship between organizational identification and task performance behavior (γ_OI→CRE→TP_; 0.183, *p* < 0.001; Z = 6.014, *p* < 0.01), because the direct effect of organizational identification on task performance is significant (0.205, *p* < 0.001).

## 6. Discussion

This study shows that transformational leadership has a significant positive impact on the employees’ organizational identification, creativity, and task performance. In the service-intensive hospitality industry where quality of service delivery relies heavily on frontline employees, effective leadership is found to play a pivotal role in improving customers’ experience (e.g., [25,26]).

The result shows that H1-1, H1-3, H2-4, H3, H4, and H5 are supported. The findings are consistent with earlier research that suggests that core transformational behavior and supportive leader behavior have a positive impact on employees’ organizational identification [69,70,71] and creativity [83]. Intellectual stimulation is found to be the only factor that enhances employees’ creativity, which has a positive influence on task performance. This finding is consistent with the Cheung and Wong’s study [80]. The finding also shows that organizational identification plays a significant mediating role in the relationship between core transformational leader behavior and creativity, and between supportive leader behavior and creativity. In addition, creativity plays a partial mediating role in the relationship between organizational identification and task performance.

Contrary to our expectations, high-performance expectations and intellectual stimulation did not emerge as significant predictors of organizational identification. This finding deviates from previous studies that reported a significant influence of high-performance expectations [72,73,74] and intellectual stimulation [78,79] on organizational identification. Given that organizational identification is an outcome from a complex process wherein employees evaluate and recognize the overall value of the organization, it can be influenced by multiple factors [117]. The effects of high-performance expectations and intellectual stimulation on organizational identification may have been shadowed by the presence of significant effects of core transformational behavior and supportive leader behavior. In other words, core transformational leadership behavior and supportive leader behavior are more critical than the other two dimensions in nurturing employees’ sense of identification with the organization. The study also finds that only intellectual stimulation has a positive effect on creativity. This result may be explained in light of the organizational culture in South Korea. The hierarchical organizational culture in Korea is found to prevent employees from openly expressing their thoughts and opinions [118]. Employees may withhold their thoughts and ideas unless the leader actively promotes “thinking outside the box”. The finding that high-performance expectations do not help increase employees’ organizational identification suggests that leaders should not use high-pressure tactics or set too high goals for the employees.

The finding that organizational identification is a full mediator in the relationship between two dimensions of leadership and creativity suggests that both core transformational leader behavior and supportive leader behavior have an indirect effect on creativity through organizational identification. In other words, employees’ creativity can be enhanced when the leader not only stimulates employees intellectually (direct effect) but also shows supportive leader behavior and core transformational behavior (indirect effect). This result highlights the important role of organizational identification in enabling leadership behaviors to bring out employees’ creativity. In addition, creativity is found to play a partial mediating role in the relationship between organizational identification and task performance.

In sum, the result of the current study shows that transformational leadership is a valued leadership style that enhances employees’ organizational identification, creativity, and task performance. Given that the hotel industry is faced with intense competition, acquiring and retaining high-quality employees are critical for distinguish the organization from others. This study suggests that leadership can make a difference to how employees perceive the organization (organizational identification), engage in the work (creativity), and perform the job (task performance). The theoretical and practical implications of the findings are discussed below.

## 7. Conclusions

The result of this study shows that transformational leadership dimensions have different effects on organizational identification and creativity. While core transformational leadership and supportive leader behavior have a direct impact on organizational identification, which consequently affects creativity, intellectual stimulation has a direct positive influence on creativity. This study helps to understand the mediating role of organizational identification and creativity.

### 7.1. Theoretical Implications

This study presented an integrated model using leadership theory and social identity theory. While some previous studies examined transformational leadership as a single dimension (e.g., [21]) or treated it as a second-order factor (e.g., [20]), this study considered multiple dimensions to capture different aspects of the leadership and their effects on employees. Using multiple dimensions is helpful for identifying different effects of the dimensions, because not all leaders possess a complete set of the leadership dimensions. The finding suggests a dynamic relationship between different dimensions of leadership and employees’ responses (organizational identification, creativity, and task performance). This will offer some implications regarding how to leverage leadership and organizational resources for goal attainment [7].

Second, this study draws a concept of organizational identification from the social identity theory to examine how four dimensions of transformational leadership influence the employees. Organizational identification is an employee’s sense of belongingness to the organization. The study shows that a leader can help employees identify themselves with the organization by focusing on core transformational leadership behavior and supportive leader behavior [96]. This finding suggests that transformational leadership plays an important role in influencing employees in terms of organizational identification.

Third, as shown in Table 5, the alternative model analysis shows that organizational identification and creativity are significant predictors of task performance in a post hoc analysis that considered a direct impact of transformational leadership on task performance. The study finds that core transformational leader behavior, supportive leader behavior, and intellectual stimulation do not directly affect task performance. This means that organizational identification and creativity play full mediating roles. High-performance expectations are found to directly affect task performance. Creativity in this case was a partial mediator. Unlike the other three dimensions, high-performance expectations were directly related to employees’ task performance.

### 7.2. Managerial Implications

This study offers several managerial implications applicable to the hotel industry.

First, this study enhances our understanding of the relationship between leadership style and employees’ responses. The dimensional approach has allowed us to pinpoint the leadership behaviors that have the most effects on the employees. Human resources are an important source of competitive advantage in the hotel industry [105,119]. The results of this study show that core transformational leadership behavior and supportive leader behavior are effective for enhancing employees’ identification with the organization. This suggests that leaders should have capabilities to articulate a vision for the organization, set the organizational goals, strategize, and create a supportive work environment where employees are given appropriate tools and resources to do the work and succeed.

Second, the finding that intellectual stimulation helps employees be creative suggests that organizations consider adopting creativity programs (e.g., divergent thinking, creative self-efficacy, and creative problem-solving skills for both leaders and employees [120,121]). These programs will help organizations find creative solutions in tackling challenges such as the economic downturn caused by the COVID-19 pandemic.

Lastly, in the post hoc analysis, it was found that high-performance expectations do not directly affect organizational identification or creativity, but directly affect task performance. This suggests a clear and direct relation between high-performance expectations and task performance. Unlike the other three leadership dimensions that influence task performance through organizational identification or creativity, high-performance expectations directly influence task performance. The finding suggests that when leaders expect employees to perform well, they will work hard. There is a caveat to consider. The literature on the theory of management by goals suggests that when a leader sets goals that are too high for employees to achieve, employees may give up. Therefore, it is important for leaders to set goals that are challenging but attainable. On the other hand, goals that are too easy to achieve will fail to motivate employees to work hard. Therefore, setting goals that are challenging yet attainable is important for enhancing employees’ job performance.

### 7.3. Limitations and Future Research Directions

The study limitations and directions for future research are discussed as follows. First, this study was conducted in the context of luxury hotels in South Korea. Therefore, the findings should not be generalized. Future research may want to do a comparative analysis by carrying out a study in other service industries (e.g., aircraft, restaurants, leisure complexes, communication services, large law firms, etc.). Second, we used the data collected from hotels managed by indigenous general managers. Future research may want to collect data from hotels managed by expatriate general managers for a comparative analysis. Finally, future studies may want to consider other exogenous variables (e.g., service-oriented culture and value), intervening variables (e.g., emotion, cognitive, and trust), and demographic variables (e.g., department, hotel grade, age, gender, etc.) that may explain organizational performance.

## Figures and Tables

**Table 1:** Demographic characteristics (*n* = 454).

Category		*n*	%
Gender	Male	212	46.7
	Female	242	53.3
Marital status	Single	234	51.5
	Married	211	46.5
	Other	3	0.6
	Missing	6	1.3
Age	20–29	146	32.2
	30–39	222	48.9
	40–49	74	16.3
	50 and more	11	2.4
	Missing	1	0.2
Educational level	High school	5	1.1
	2-year college	120	26.4
	4-year university	276	60.8
	Graduate school or more	50	11.0
	Missing	3	0.7
Position	Staff	240	52.9
	Manager	143	31.5
	Department head (Team leader level)	63	13.9
	Executives	8	1.8
Department	Front	55	12.1
	Room	75	16.5
	Food and Beverage	81	17.8
	Culinary	59	13.0
	Management	89	19.6
	Other	84	18.5
	Missing	11	2.4
Duration of working experience	Four and under	195	43.0
	Five and over	259	57.0

**Table 2:** Measurement model.

Items	Standardized Factor Loadings	Cronbach’s Alpha	CR ^a^	AVE ^b^
**Organizational identification**		0.916	0.947	0.857
This hotel has a deep meaning for me. ^#^				
I have a strong sense of identifying with this hotel.	0.935			
I have a strong sense of belonging to this hotel.	0.926			
I have special feelings to this hotel.	0.915			
**Creativity**		0.929	0.949	0.824
I will suggest new ways to achieve goals or objectives.	0.897			
I will come up with new and practical ideas to improve performance.	0.928		0.755 *	0.794 *
I will search out new technologies, processes, techniques, and/or product ideas.	0.893		0.726 *	0.723 *
I will suggest new ways to increase quality.	0.911		0.779 *	0.756 *
**Core transformational leader behavior**		0.886	0.930	0.815
Articulate a vision.	0.901			
Provide an appropriate model.	0.918			
Facilitate the acceptance of group goals.	0.889			
**High-performance expectations**				
Makes it clear to me that she or he expects me to give 110% all of the time.	1.000			
Insists on only the best performance. ^#^				
**Intellectual stimulation**		0.861	0.915	0.783
Challenges me to think about old problems in new ways. ^#^				
Asks questions that prompt me to think about the way I do things.	0.890			
Has stimulated me to rethink the way I do some things.	0.885			
Has ideas that have challenged me to reexamine some of my basic assumptions about my work.	0.879			
**Supportive leader behavior**		0.920	0.943	0.806
Acts considering my personal feelings.	0.885			
When I am difficult, I can rely on my owner.	0.903			
Is friendly and approachable.	0.898			
Considers my personal specific situation (or business).	0.905			
Is not difficult to communicate with my owner. ^#^				
**Task performance**		0.928	0.946	0.778
Performs his/her job well.	0.870			
Adequately completes assigned duties.	0.908			
Fulfills responsibilities specified in job description.	0.862			
Performs tasks that are expected of him/her.	0.897			
Meets formal performance requirements of the job.	0.871			

^a^ CR (Composite Reliability), ^b^ AVE (Average Variance Extracted); * *p* < 0.01; ^#^ Items were deleted during the measurement model analysis.

**Table 3:** Fornell–Larcker criterion and correlation matrix.

Constructs	1	2	3	4	5	6	7
1. Core transformational leader behavior	**0.903**						
2. High-performance expectations	0.607	**-**					
3. Supportive leader behavior	0.528	0.259	**0.898**				
4. Intellectual stimulation	0.558	0.377	0.789	**0.885**			
5. organizational identification	0.483	0.313	0.490	0.470	**0.926**		
6. Creativity	0.419	0.321	0.385	0.450	0.592	**0.908**	
7. Task performance	0.336	0.366	0.192	0.255	0.438	0.512	**0.882**
Mean							
SD							

Note: All coefficients are significant at *p* < 0.01. Note: Bold numbers indicate the square root of AVE.

**Table 4:** Heterotrait–Monotrait ratio (HTMT).

Constructs	1	2	3	4	5	6	7
1. Core transformational leader behavior							
2. High-performance expectations	0.646						
3. Supportive leader behavior	0.583	0.271					
4. Intellectual stimulation	0.636	0.406	0.886				
5. organizational identification	0.534	0.326	0.535	0.530			
6. Creativity	0.460	0.333	0.417	0.504	0.641		
7. Task performance	0.370	0.379	0.207	0.285	0.473	0.551	

Numbers: total sample/males/females.

**Table 5:** Structural estimates (PLS).

		Proposed Model					Alternative Model				
	Paths	Estimate	t	*p*	f^2^	Results	Estimate	t	*p*	f^2^	Results
H1-1	CTL → OI	0.257	4.517	0.000	0.046	Supported	0.257	4.532	0.000	0.046	*p* < 0.001
H1-2	HPE → OI	0.051	0.933	0.351	0.002	Not supported	0.051	0.925	0.355	0.002	n.s
H1-3	SLB → OI	0.261	3.157	0.002	0.035	Supported	0.261	3.169	0.002	0.035	*p* < 0.01
H1-4	IS → OI	0.102	1.108	0.268	0.005	Not supported	0.102	1.117	0.264	0.005	n.s
H2-1	CTL → CRE	0.063	1.072	0.284	0.003	Not supported	0.063	1.074	0.283	0.003	n.s
H2-2	HPE → CRE	0.070	1.412	0.158	0.005	Not supported	0.070	1.408	0.159	0.005	n.s
H2-3	SLB → CRE	−0.077	1.218	0.223	0.003	Not supported	−0.077	1.212	0.225	0.003	n.s
H2-4	IS → CRE	0.228	3.478	0.001	0.029	Supported	0.228	3.481	0.001	0.029	*p* < 0.01
H3	OI → CRE	0.470	9.717	0.000	0.252	Supported	0.470	9.716	0.000	0.252	*p* < 0.001
H4	OI → TP	0.207	3.882	0.000	0.039	Supported	0.205	3.524	0.000	0.000	*p* < 0.001
H5	CRE → TP	0.390	7.946	0.000	0.139	Supported	0.361	7.287	0.000	0.037	*p* < 0.001
	CTL → TP						0.024	0.375	0.708	0.005	n.s
	HPE → TP						0.203	3.721	0.000	0.000	*p* < 0.001
	SLB → TP						−0.101	1.480	0.139	0.034	n.s
	IS → TP						−0.014	0.212	0.832	0.118	n.s
		R^2^	Q^2^				R^2^	Q^2^			
	OI	0.316	0.296				0.316	0.296			
	CRE	0.400	0.233				0.400	0.233			
	TP	0.290	0.110				0.400	0.141			
	SRMR	0.050					0.041				
	VIF	1.646–2.961					1.462–3.037				

CTL: Core transformational leader behavior, HPE: High-performance expectations, SLB: Supportive leader behavior, IS: Intellectual stimulation, OI: Organizational identification, CRE: Creativity, TP: Task performance, VIF: Variance inflation factor, SRMR: Standardized root mean square residual, n.s.: non-significant.

**Table 6:** Mediation test (PLS).

Paths	Direct Effect	Indirect Effect	CI [LLCI, ULCI]	Z-Value	Mediating Role
CTL → OI → CRE	0.063 ^n.s.^	0.121 ***	[0.067, 0.180]	3.572 ***	Full
HPE → OI → CRE	0.070 ^n.s.^	0.024 ^n.s.^	[−0.025, 0.074]		-
SLB → OI → CRE	−0.077 ^n.s.^	0.123 **	[0.052, 0.210]	2.994 **	Full
IS → OI → CRE	0.228 **	0.048 ^n.s.^	[−0.043, 0.131]		-
OI → CRE → TP	0.205 ***	0.183 ***	[0.134, 0.245]	6.014 ***	Partial

Note: ** *p* < 0.05, *** *p* < 0.01, CI: Confidence interval, LLCI: Lower limit confidence interval, ULCI: upper limit confidence interval, n.s. = non-significant.

## Data Availability

The data presented in this study are available on request from the corresponding.
